# Comparison of Intrauterine Suitable Balloon and Foley Balloon in the Prevention of Adhesion after Hysteroscopic Adhesiolysis

**DOI:** 10.1155/2018/9494101

**Published:** 2018-11-08

**Authors:** Ru Zhu, Hua Duan, Lu Gan, Sha Wang

**Affiliations:** ^1^Department of Minimally Invasive Gynecology, Beijing Obstetrics and Gynecology Hospital, Capital Medical University, Beijing 100006, China; ^2^Department of Obstetrics and Gynecology, Anqing Hospital Affiliated to Medical University of Anhui, Anqing 246003, China

## Abstract

**Objective:**

To compare the effect of intrauterine suitable balloon (ISB) and Foley balloon (FB) in the prevention of adhesion after hysteroscopic adhesiolysis in patients with intrauterine adhesions (IUAs).

**Methods:**

A retrospective study was conducted on 150 women with moderate and severe IUAs, who underwent hysteroscopic adhesiolysis. According to the postoperative placement of the ISB or FB, the cohort was divided into the ISB group and the FB group. A second-look hysteroscopy was performed at 3 months postsurgery. The scoring system proposed by the American Fertility Society (AFS) was used to evaluate the adhesion during hysteroscopy. Subgroup analysis was carried out based on the degree of IUAs.

**Results:**

(1) In the ISB group, only 25% (19/76) women presented adhesion reformation after surgery, while, in the FB group, the adhesion reformation was observed in 35.1% (26/74) patients; however, the difference was not statistically significant (*P*>0.05). Subsequently, the adhesion reformation rate (29.5%, 13/44) after surgery with an ISB for severe intrauterine adhesions was significantly lower as compared to that (53.6%, 15/28) with FB (*P*<0.05). (2) In the ISB group, the reduction in the adhesion score after surgery was 8 points, which was significantly higher than the 7 points in the FB group (*P*<0.05).

**Conclusion:**

The ISB is better than the FB in preventing the adhesion reformation and reducing the AFS score after hysteroscopic adhesiolysis in severe IUAs. Also, it can effectively prevent the adhesion reformation in severe IUAs with a similar effect on moderate IUAs.

## 1. Introduction

Intrauterine adhesions (IUAs) occur due to the various causes of damage to the basal layer of the endometrium and intramural uterine adhesion that disrupts the uterine anatomy. Consequently, a series of clinical symptoms, including abnormal menstrual conditions (especially hypomenorrhea and amenorrhea), infertility, recurrent pregnancy loss, and abnormal placental location of placenta implantation or placenta previa are observed [1]. The incidence of IUAs in abnormal menstruation and infertility is 2.8–45.5% [2], which severely affects the menstrual physiology and reproductive function in women.

The standard method of treatment of IUAs is transcervical resection of adhesion (TCRA), wherein the high recurrence rate of postoperative adhesion is the primary challenge. In the case of patients with severe IUAs, the recurrence rate of postoperative adhesions is up to 62.5% [1]. Presently, several auxiliary measures prevent the recurrence of adhesion after TCRA, including physical barriers, biogel, estrogen, and amniotic membrane. Nevertheless, a universally recognized effective standard preventive strategy is yet lacking [3, 4].

**Figure 1:**
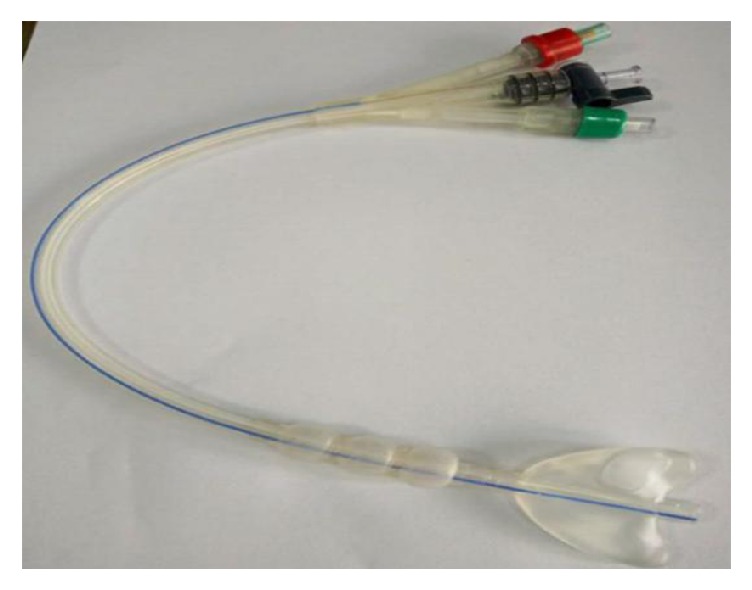
Intrauterine suitable balloon.

The leading physical barriers that can prevent the recurrence of adhesion after TCRA include intrauterine device and balloon device. Although the contraceptive device is used for preventing the recurrence of postoperative adhesion, disadvantages such as the limited area of isolation and the induction of local inflammatory response are encountered [5]. Thus, the use of Foley balloon (FB) is reported to prevent the formation of adhesion, as well as improve the volume of menstruation [6]. Nonetheless, a definite defect is noted in the FB. The shape of the balloon does not match that of the uterine cavity, and hence it cannot isolate the two sides of the uterine cavity and uterine horn accurately. The local pressure might affect the blood supply of the endometrium [7]. Recently, a “heart” type of intrauterine balloon was used for preventing the recurrence of adhesions; this effect was similar to that of an intrauterine device (IUD), albeit without any statistical significance [8]. Therefore, finding an optimal isolation barrier after TCRA is an urgent requisite in the clinical setting. Herein, we designed a novel intrauterine suitable balloon (ISB) (Figure 1, patent number: 201420679083.7), which is triangular with slight protuberance at the corners, matching to that of the uterine cavity. The device can effectively isolate the two sides of the uterine cavity and bilateral uterine horn, and it also consists of a drainage channel and an injection channel. The channels can sufficiently drain the exudate from the uterine cavity and inject the antiadhesion drugs into the uterus or promote the growth of the endometrium. In addition, a balloon was present in the cervical canal, which prevented the outflow of drugs into the uterine cavity. Theoretically, it can further reduce the recurrence rate of adhesions after TCRA.

In this retrospective cohort study, we compared the effect of intrauterine balloon and FB after TCRA on preventing the postoperative recurrence of adhesions.

## 2. Materials and Methods

### 2.1. Patients

This retrospective cohort study screened the data from all cases obtained from the electronic medical record database. From January 2013 to December 2017, patients with IUAs, who underwent TCRA operation at the Department of Gynecology Minimally Invasive Center of Beijing Obstetrics and Gynecology Hospital Affiliated to Capital Medical University, were selected as the research subjects using an ISB and FB as preventive measures. A total of 150 surgical patients with moderate or severe uterine adhesion were admitted every year at our center in the last 5 years. This study was approved by the Medical Ethics Committee of Beijing Obstetrics and Gynecology Hospital. The inclusion criteria for the patients were as follows: (1) the age was 18–40 years; (2) the diagnosis of hysteroscopy in the outpatient department was moderate and severe IUAs; (3) endocrines and ovulation were normal. The exclusion criteria for the patients were as follows: (1) surgery failed to completely restore the normal form of the uterine cavity; (2) no hysteroscopy was conducted in 3 months postoperative; (3) there was TCRA operation history; (4) lack of hormone-controlled cycles and antibiotic prophylaxis existed.

### 2.2. Diagnosis and Classification of IUAs

The diagnosis and scoring of IUAs was based on the 1988 American Fertility Society (AFS) standard [9]. According to the nature of adhesions, scope of adhesions, and menstrual mode, a quantitative scale was designed: 1–4 indicates mild, 5–8 indicates moderate, and 9–12 indicates severe. All the scores were derived from the original data.

### 2.3. Methods of Operation and Postoperative Treatment

Hysteroscopic lysis was performed by an experienced endoscopic surgeon under general anesthesia for all patients. The surgical equipment and instruments utilized were as follows: Olympus S70 operation hysteroscope series equipment, operation hysteroscope, and matched 27Fr passive continuous perfusion bipolar electroscope. The motor power was set at 320 W/160 W, and the perfusion medium was normal saline. Tracheal intubation plus venous-combined general anesthesia was used for surgical anesthesia. The routine cervix preconditioning (400 *μ*g misoprostol was placed in the posterior vagina) was performed at a late stage before the operation. The TCRA operation was guided by transabdominal ultrasonography. In hysteroscopy, the comprehensive observation of uterine cavity morphology and adhesion, degree of tissue adhesion, and joint ring electrode excision of scar tissue by needle electrode separation were under intensive focus to assess the protective role of the residual endometrial tissue until the uterine cavity returned to normal morphology, free of adhesions. Also, bilateral uterine horn and tubal opening were either visible or invisible.

**Figure 2:**
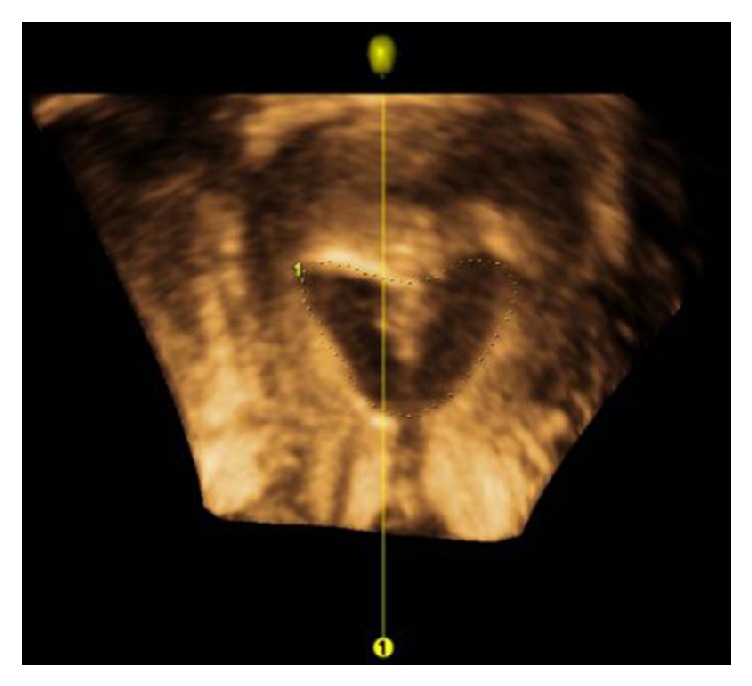
Ultrasound images of the intrauterine cavity in the ISB.

The choice of balloons depends primarily on the preferences of the surgeons. After the recovery of the uterine cavity, the gas in the ISB was aspirated to exert a negative pressure, wrapped around the lumen, and then rotated along the cervical canal into the uterine cavity. Subsequently, 3-4 mL saline was injected into the balloon that was fully expanded in the intrauterine (Figure 2). The balloon catheter was connected to the drainage bag device. Then, the ISB was removed 5–7 days after the operation by aspirating the saline and withdrawing the balloon. The top part of the FB was cut off before placement. The method of placing and pulling out the FB was similar to that of the ISB, and the complications were recorded.

The two groups were administered the same hormonally controlled cycles (n=3) on the second day after operation, based on the previous experience [10, 11]. Each cycle was described as follows (oral administration): 4 mg/d estradiol valerate tablets (Progynova; Bayer; Delpharm Lille S.A.S) for 21 days and 20 mg/d dydrogesterone tablets (Abbott Biologicals B.V) for the following 10 days [4]. All subjects were injected cefmetazole sodium (Sichuan Hexin Pharmaceutical Co. Ltd, Sichuan, China) for 7 days.

### 2.4. Follow-Up

The patients were followed up in the outpatient clinic for 1 and 3 months. Hysteroscopy was performed with a 4.5-mm hysteroscope using normal saline as the perfusion fluid. The procedure was performed by a full-time experienced hysteroscopy evaluator in the outpatient clinic after the initial operation. The evaluator was unaware of the type of balloon used during the initial procedure for observing the adhesion and recording the AFS score [6, 12].

### 2.5. Statistical Methods

SPSS 22 software (IBM, Armonk, NY, USA) was used for statistical analysis. The normal distribution measurement data were represented as $\bar{x}\pm SD$ (mean and standard deviation), and the two groups were compared using independent sample t-test. The nonnormal distribution measurement data were expressed as median, and the comparison between the groups was performed using Mann–Whitney U test. The qualitative data were statistically described by the rate (ratio); chi-square test was used for the comparison of disordered data, and the Mann–Whitney U test was used for the comparison of ordinal data between the groups. All the statistical tests were verified by bilateral tests, and* P*<0.05 indicated statistical significance. The data were represented using GraphPad Prism 6 software (IBM).

## 3. Results

### 3.1. Baseline Characteristics of the Two Groups of Patients

**Figure 3:**
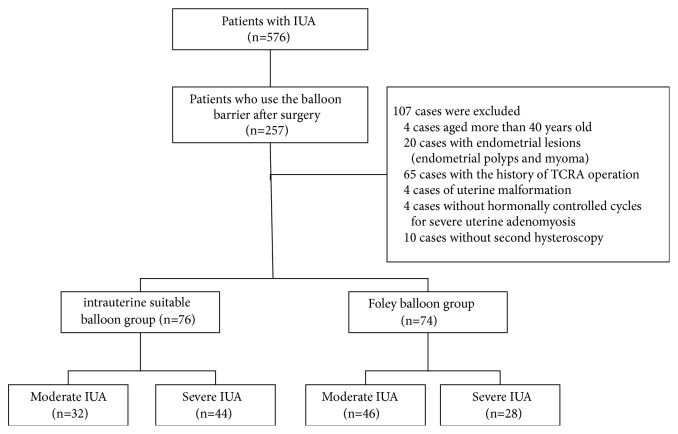
Schematic of case screening.

From January 2013 to December 2017, 576 cases of moderate-to-severe adhesion were obtained from the Department of Gynecology Minimally Invasive Center of Beijing Obstetrics and Gynecology Hospital Affiliated to Capital Medical University. Among these, 257 cases were treated with an ISB or FB postoperatively. A total of 107 cases were excluded (those who did not fulfill the inclusion and exclusion criteria), and 150 cases were included in the current analysis. IUAs were mainly caused by pregnancy-related curettage (90.7%, 136/150). The ISB group comprised 76 cases: 32 cases had moderate IUAs, and 44 cases had severe adhesions IUAs. The FB group comprised 74 cases: 46 cases had moderate IUAs, and 28 cases showed severe IUAs. The case screening process is illustrated in Figure 3. The clinical features of the two groups are listed in Table 1. No statistically significant differences were observed in the other basic features except for the ratio of the adhesion degree in the two groups (*P*<0.05).

### 3.2. Recurrence of Postoperative Adhesion in the Two Groups

The recurrence rate of postoperative adhesion (25%, 19/76) in the ISB was lower than that in the FB group (35.1%, 26/74), albeit not significantly (*P*=0.176). The recurrence rate of postoperative intrauterine balloon in severe IUAs (29.5%, 13/44) was significantly lower than that of the FB (53.6%, 15/28) (*P*=0.041; Table 2).

### 3.3. Reduction in AFS Scores (Preoperative Score Minus Postoperative Score) in Both Groups of Patients before and after Operation

The postoperative reduced AFS score in the ISB group (8 points) was significantly lower than that of the FB group (7 points) (*P*=0.016). Also, statistically significant differences were observed in the reduction of adhesion score among severe IUAs (Table 2).

## 4. Discussion 

In this study, we compared the effects of different balloon devices on the prevention of recurrence in patients with moderate and severe adhesions. The results showed that the recurrence rate of the ISB after TCRA was lower than that of the FB, especially in patients with severe IUAs. The reduction in the AFS score with ISB was significantly higher than that of the FB postoperatively.

The standard treatment method for IUAs is TCRA, and some auxiliary measures are used to prevent the recurrence of adhesion after operation [13]. Thus, FB is recommended in order to prevent the recurrence of adhesion after IUAs as it can effectively reduce the recurrence of adhesion post-TCRA [14], restore the morphology of the uterine cavity, and improve the menstrual status [15]. However, the structure of the FB does not conform to the shape of the uterine cavity, and hence cannot reduce the recurrence of adhesion after the operation [10]. In addition to the use of Foley balloon, hormone therapy is also used in the study. Nevertheless, the recurrence rate is still 35.1%, which is similar to the results of the present study (33.3%, 4/12) [14]. The recurrence rate of severe adhesion patients is 53.6%, which is similar to the results (48%) of a previous study [16] that utilized a fresh amnion graft over an inflated Foley catheter to prevent the recurrence of adhesion.

In this study, two groups of balloon devices were partially similar in preventing adhesion with respect to physical barrier isolation. An equivalent volume of saline was administered in the balloon, the same retention time was observed, the drainage pipe drained off the exudate, and the top of the FB catheter was cut off in order to reduce the influence of the duct on the uterine cavity. After 3 months, the results of the two sets of devices varied significantly, especially in the case of patients with severe IUAs. Therefore, we speculated that the use of the intrauterine suitable balloon after TCRA reduced the recurrence of adhesions rather effectively.

The location of IUAs was closely related to the recurrence of postoperative adhesion that is mostly seen in the lower segment of the uterus, the peripheral, and the corner of the uterus [17]. In order to isolate the uterine cavity wounds effectively, the researchers [2] attempted to use a “heart”-type balloon device to prevent the recurrence after adhesion surgery. Recently, a prospective randomized controlled study on intrauterine [8] reported that of the 82 cases of patients with moderate-to-severe postoperative uterine balloon type, patients with severe adhesions accounted for 35.4% (29/82); the postoperative recurrence rate was 30% (29/82), while the effect of prevention did not vary significantly. In this study, 76 patients with severe IUAs (57.9%) were treated with an intrauterine conformable balloon after the operation, and the total recurrence rate was only 25%. The two kinds of balloon barriers exhibited similarities in appearance, and the placement time was similar to that of the postoperative intrauterine preservation [8]. However, the recurrence rate of adhesions after intrauterine balloon type was low, especially in patients with severe adhesion. This phenomenon might be attributed to the ISB with uterine cavity drainage catheter, which can drain the exudate early after the operation and reduce the formation of adhesions. The shape of the ISB was similar to the normal uterine cavity, especially uterine horn. However, due to the lack of direct comparison of these two types of balloon devices, a multicenter, prospective, randomized, controlled study is required in the later period.

The ISB can significantly reduce the postoperative AFS score of moderate-to-severe patients and effectively reduce the recurrence rate of patients with severe adhesions, primarily due to its suitable shape that matches the uterine cavity. The three-dimensional ultrasound images (Figure 3) displayed the state of ISB in the uterine cavity that can effectively separate the walls of the uterine cavity and the bilateral angle of the uterus. In addition, the ISB was flat with a uniform pressure on the anterior and posterior wall of the uterus. It would not cause excessive pressure in the local area and affect the blood supply of the endometrium.

This study, for the first time, introduced the new type of intrauterine balloon that was similar to the traditional FB in preventing the postoperative recurrence of TCRA. The two groups of cases were assessed via stringent screening, and a large number of cases were included. To evaluate the preventive effect, we not only compared the recurrence of adhesions but also quantitatively analyzed the difference in the degree of AFS adhesion decline after using the two auxiliary measures, which increased the efficiency of research. Moreover, the present study excluded the patients with TCRA treatment history. Most patients underwent initial surgery in other hospitals, and hence, the initial adhesions and recurrent factors after multiple operations were unclear [18]. Considering that the degree of IUAs is the primary factor influencing the postoperative efficacy [12, 18], a subgroup analysis was conducted according to the degree of IUAs, followed by the stratification of the patients with different degrees of adhesion.

Nevertheless, the inadequacy of the present study was the retrospective design, and the surgeon did not follow random principles for the selection of the two balloon barriers. However, the baseline characteristics of two groups did not differ significantly. The inclusion and exclusion criteria for the recruitment of research subjects ensured adequate representation of the sample. Furthermore, the surgeon in our center had abundant experience in TCRA operation. The relevant clinical information was obtained from the electronic medical record database. The diagnostic scoring method for IUAs referred to the AFS IUAs score standard. In addition, the stratified analysis was carried out according to the degree of adhesion in the analysis stage of the data, which reduced the study bias. Therefore, further studies are essential to substantiate the current findings.

## 5. Conclusions

The results of this retrospective study showed that the effect of the ISB to prevent the recurrence of IUAs and reduce AFS was significantly better than the FB for severe IUAs. However, the advantage of the treatment for moderate adhesions was not obvious. Thus, a rigorous prospective randomized controlled trial is imperative in order to observe the effect of preventing intrauterine horn adhesion and follow-up of the outcome of birth.

## Figures and Tables

**Table 1:** Demographic and clinical parameters of the patient.

Variables	FB group	ISB group	*P* value
Number	74	76	
Age (years)^a^	30.9 ± 3.6	31.2 ± 4.3	0.566
Times of pregnancy^b^	2 (1–3)	2 (1–3)	0.435
Childbirth^c^	8 (10.8%)	17 (22.4%)	0.058
Body Mass Index^a^	21.8 ± 3.7	22.5 ± 4.1	0.290
Menstrual pattern^c^			0.997
Amenorrhea	7 (9.5%)	7 (9.2%)	
Hypomenorrhea	59 (79.7%)	61 (80.3%)	
Normal menstruation	8 (10.8%)	8 (10.5%)	
Etiological type^c^			0.256
Artificial abortion	21 (28.4%)	20 (26.3%)	
Curettage in early pregnancy	39 (52.7%)	36 (47.4%)	
Curettage in the middle and late pregnancy^c^	11 (14.9%)	9 (11.8%)	
Unexplained reasons	1 (1.4%)	5 (6.6%)	
Other	2 (2.7%)	6 (7.9%)	
Number of times of pregnancy loss^c^			0.534
0	39 (52.7%)	43 (56.6%)	
1	22 (29.7%)	23 (30.3%)	
≥2	13 (17.6%)	10 (13.2%)	
Number of times of intrauterine operation^b^	2 (1–3)	2 (1–2.75)	0.390
Endometrial thickness (mm)^a^	5.8 ± 2.1	5.9 ± 2.1	0.839
Uterine volume (cm^3^)^a^	44.7 ± 14.3	49.2 ± 20.5	0.123
Degree of adhesion^c^			0.014
Moderate adhesion	46 (62.2%)	33 (42.1%)	
Severe adhesion	28 (37.8%)	44 (57.9%)	
Preoperative adhesion type^c^			0.333
Mixed type	42 (56.8%)	49 (64.5%)	
Peripheral type	32 (43.2%)	27 (35.5%)	
Preoperative intrauterine depth^a^	7.7 ± 0.9	7.7 ± 0.9	0.976

^a^Mean ± SD,* t*-test

^b^Median (95% confidence interval), Mann–Whitney *U* test

^c^Number (percentage), *χ*^2^ test

**Table 2:** Comparison of AFS and recurrence of adhesion in the two groups.

	FB	ISB group	*P* value
Preoperative AFS (score)^b^			
All	8 (7–10)	10 (7–10)	0.068
Severe IUAs	10 (10–10)	10 (10–10)	0.725
Moderate IUAs	7.5 (6–8)	7 (6–8)	0.208
Postoperative AFS^b^			
All	0 (0–1.25)	0 (0–3.75)	0.205
Severe IUAs	5.0 (0–7.75)	0 (0–5)	0.035
Moderate IUAs	0 (0–1.25)	0 (0–0)	0.667
AFS reduction^b^			
All	7 (3.75–8)	8 (5–10)	0.016
Severe IUAs	5.0 (2.5–10.0)	10.0 (5.0–10.0)	0.033
Moderate IUAs	7.0 (4.5–8.0)	7.0 (5.0–7.75)	0.595
Recurrence (n)^c^			
All	26 (35.1%)	19 (25.0%)	0.176
Severe IUAs	15 (53.6%)	13 (29.5%)	0.041
Moderate IUAs	11 (23.9%)	6 (18.8%)	0.587

All = severe and moderate IUA

^b^Median (95% confidence interval), Mann–Whitney *U* test

^c^Number (percentage), *χ*^2^ test

## Data Availability

The data used to support the findings of this study are available from the corresponding author upon request.
